# The female reproductive tract microbiotas, inflammation, and gynecological conditions

**DOI:** 10.3389/frph.2022.963752

**Published:** 2022-08-09

**Authors:** Mahsa Gholiof, Emma Adamson-De Luca, Jocelyn M. Wessels

**Affiliations:** ^1^Department of Obstetrics and Gynecology, McMaster University, Hamilton, ON, Canada; ^2^AIMA Laboratories Inc., Hamilton, ON, Canada

**Keywords:** female reproductive tract (FRT), inflammation, immune response, microbiome, gynecological diseases

## Abstract

The intricate interactions between the host cells, bacteria, and immune components that reside in the female reproductive tract (FRT) are essential in maintaining reproductive tract homeostasis. Much of our current knowledge surrounding the FRT microbiota relates to the vaginal microbiota, where ‘health’ has long been associated with low bacterial diversity and *Lactobacillus* dominance. This concept has recently been challenged as women can have a diverse vaginal microbial composition in the absence of symptomatic disease. The structures of the upper FRT (the endocervix, uterus, Fallopian tubes, and ovaries) have distinct, lower biomass microbiotas than the vagina; however, the existence of permanent microbiotas at these sites is disputed. During homeostasis, a balance exists between the FRT bacteria and the immune system that maintains immune quiescence. Alterations in the bacteria, immune system, or local environment may result in perturbances to the FRT microbiota, defined as dysbiosis. The inflammatory signature of a perturbed or “dysbiotic” FRT microbiota is characterized by elevated concentrations of pro-inflammatory cytokines in cervical and vaginal fluid. It appears that vaginal homeostasis can be disrupted by two different mechanisms: first, a shift toward increased bacterial diversity can trigger vaginal inflammation, and second, local immunity is altered in some manner, which disrupts the microbiota in response to an environmental change. FRT dysbiosis can have negative effects on reproductive health. This review will examine the increasing evidence for the involvement of the FRT microbiotas and inflammation in gynecologic conditions such as endometriosis, infertility, and endometrial and ovarian cancer; however, the precise mechanisms by which bacteria are involved in these conditions remains speculative at present. While only in their infancy, the use of antibiotics and probiotics to therapeutically alter the FRT microbiota is being studied and is discussed herein. Our current understanding of the intimate relationship between immunity and the FRT microbiota is in its early days, and more research is needed to deepen our mechanistic understanding of this relationship and to assess how our present knowledge can be harnessed to assist in diagnosis and treatment of gynecologic conditions.

## Introduction

**Figure 1:**
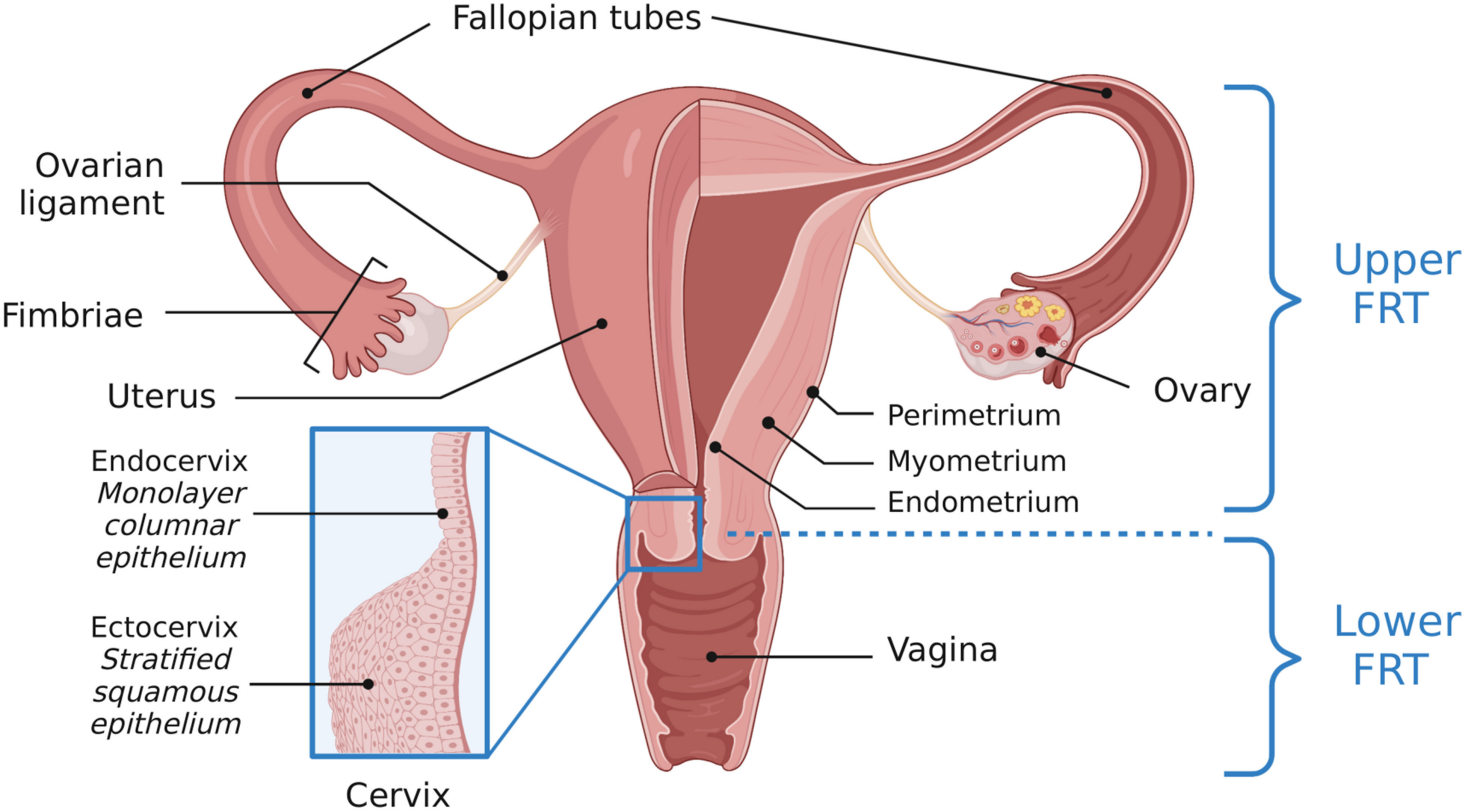
Anatomy of the female reproductive tract. A visual depiction of the female reproductive tract (FRT), further subdivided into the upper and lower reproductive tract. The lower reproductive tract consists of the vagina and ectocervix, lined by a stratified squamous epithelium. The endocervix, lined by a monolayer columnar epithelium, and the uterus, Fallopian tubes and ovaries comprise the upper reproductive tract. Adapted from “Female Reproductive System Anatomy,” by BioRender.com (2022). Retrieved from https://app.Biorender.com/Biorender-templates.

The female reproductive tract (FRT) contains a complex ecosystem including host cells, immune components, microorganisms, and metabolites. The FRT consists of a series of connected tissues and organs including the vagina, cervix, uterus, two Fallopian tubes, and two ovaries. These organs are further sectioned into the upper and lower FRT. The lower FRT is comprised of the vagina and ectocervix. The upper FRT refers to the endocervix, uterus, Fallopian tubes, and ovaries (Figure 1). Epithelial cells act as a barrier between “outside” and “inside.” The lower FRT has a protective stratified squamous epithelial barrier. The structures of the upper FRT are lined by a columnar epithelial monolayer (1, 2). The reproductive microbiotas lie in close proximity and oppose the epithelial cells lining the FRT. The human vaginal microbiota is well-characterized and commonly associated with *Lactobacillus* dominance, although evidence of polymicrobial vaginal microbiotas not associated with any clinical symptoms challenges the concept of what constitutes a “healthy” or “normal” vaginal microbiota (3). Conversely, the other reproductive tract microbiotas including the Fallopian tubes, ovaries, and endometrium, are not as thoroughly characterized but have distinct compositions (4–9). Additionally, inter-individual variation in microbial communities exists and may be influenced by different factors, such as living habits, ethnicity, diet, and immunity (10).

As a mucosal tissue, the FRT is crucial in the induction and function of immune responses. Toll-like and nod-like receptors expressed by cells in the vagina recognize pathogenic microbial species and mount an immune response to avoid infection (11). Within the FRT microenvironment, sex hormones (i.e., estrogen and progesterone), the vaginal microbiota, and hormonal contraceptives all communicate with the immune system (1). Interactions between components of the FRT microenvironment are important in genital tract homeostasis and a change in their balance may contribute to various pathologies, compromising reproductive and gynecological health. In fact, dysbiosis in the FRT coincides with genital tract infection, poor pregnancy outcome, and gynecologic cancer (10). Vaginal dysbiosis is characterized by loss of *Lactobacillus* dominance and an increase in microbial diversity that disturbs eubiosis; the interspecies balance within a host's microbiota (12).

The FRT is not only home to bacteria but has a distinct virome [viruses; reviewed in (13, 14)] and mycobiome [fungi; reviewed in (15)], however the present review will focus solely on the reproductive tract microbiotas (bacteria). Herein, we aim to summarize the current literature describing the influence of the FRT microbiota on inflammation and the consequences of this inflammatory response. We also explore experimental therapeutics. Additionally, while much of the existing literature emphasizes the role of the FRT microbiotas and inflammation in pregnancy [as reviewed elsewhere in (16, 17)] and sexually transmitted infections (STIs) [reviewed in (18, 19)], this review will mainly focus on how the microbiotas might contribute to different gynecological conditions *via* their interaction with the host immune system.

## Composition of the female reproductive tract microbiotas

### The vaginal microbiota

Studies report that the human body hosts around 4 × 10^13^ bacteria, encompassing thousands of different bacterial species, with each site of the body having its own unique complement of microorganisms and communities: its microbiota (20). The human microbiome has been shown to play an important role in health and disease (21, 22). Much of our knowledge of the FRT microbiota relates to the vaginal microbiota, the native bacteria found lining the vaginal epithelial cells that exists in a symbiotic relationship with its host. The vagina contains ~10^10^-10^11^ bacteria and therefore has the greatest biomass of any component of the FRT (23). The bacteria are found within the mucus layer that coats the surface of the genital tract epithelial cells, which act as the first line of host defense. These epithelial cells express pattern recognition receptors (PRRs) capable of identifying microorganisms, and recruiting inflammatory factors and cells as necessary to ward off microbial invasion (24, 25), as will be discussed in greater detail below.

Although the vagina is a mucosal tissue, the microbiota at this location is very different from that found lining the mucosal surface of the gut. Gut health is associated with high microbial diversity and loss of microbiota diversity leads to intestinal dysbiosis (26), whereas in the vagina an absence of *Lactobacillus* dominance (and therefore increased microbial diversity) leads to dysbiosis (12). Vaginal “health” is associated with low bacterial diversity, and dominance of *Lactobacillus* species. Indeed, several community state types have been reported, the majority of which are dominated by lactobacilli. At least five community state types (CST) exist in the vaginal microbiota of reproductive age women (3, 27). CST-I is dominated by *Lactobacillus crispatus*; CST-II by *L. gasseri*; CST-III by *L. iners*; and CST-V by *L. jensenii*. CST-IV is more polymicrobial, but can be dominated by anaerobic bacteria, have partial aerobic bacteria (classified as aerobic vaginitis, AV) or contain a small proportion of *Lactobacillus* spp., essentially making CST-IV a *Lactobacillus*-deficient vaginal microbiota (28). Various studies have demonstrated the human vaginal microbiota usually contains one of the four CSTs dominated by *Lactobacillus* species which thrive in the glycogen-rich vaginal environment and protect their host against pathogens by producing bacteriocins, hydrogen peroxide, lactic acid or by competitively excluding other bacteria (23, 28). The role of hydrogen peroxide in FRT has been challenged by *in vivo* studies (29); the hypoxic nature of the cervicovaginal environment does not support the production of oxygen in large amounts which is required for hydrogen peroxide production to achieve antimicrobial activity in FRT (30–32). Moreover, hydrogen peroxide levels have been reported using *in vitro* conditions that do not appropriately represent the cervicovaginal environment (30, 33). Therefore, future research should focus on the antimicrobial mechanism of *Lactobacillus* spp. that is translatable *in vivo*. The fifth community (CST-IV) is dominated by anaerobic bacteria (28). Lactobacilli are Gram-positive, anaerobic, rod-shaped bacteria that produce lactic acid by metabolizing different glycogen breakdown products in the vagina. The production of lactic acid results in a vaginal pH range of 2.8–4.2 (34–36). The resultant low vaginal pH (34) hinders growth of potentially harmful bacteria (34, 37, 38). Another method by which lactobacilli offer protection from other potential pathogens is through competition for space by adhering to the vaginal epithelial cells and producing compounds that are toxic to other bacteria, effectively making it more difficult for other species to thrive (39). As a result, lactobacilli are often abundant in the vagina. However, studies have shown that lactobacilli have different protective capacities; *L. crispatus, L. gasseri, L. iners*, and *L. jensenii* have been reported as the most common bacterial species in the vagina (40), but *L. iners* appears to provide a lesser degree of protection compared to the other types of lactobacilli (41). This suggests that even if a vaginal microbiota is dominated by lactobacilli, the level of protection likely differs depending on which lactobacilli are present [reviewed in (42)]. Furthermore, the concept that *Lactobacillus* dominance is associated with health has been challenged as there are asymptomatic, “healthy” women with a polymicrobial vaginal microbiota or one dominated by anaerobic bacteria. Indeed, the vaginal microbiota can vary in women of different ethnic, geographic, and sociodemographic backgrounds (3, 43), and its composition depends on a variety of factors including host genetics, physiology, and behavior [reviewed in (42)]. For example, a genetic polymorphism in TLR4, the cell-surface receptor for innate immune recognition of Gram-negative bacteria, or the anti-inflammatory mediator IL-1 receptor antagonist influences the composition of the vaginal microbiota (44, 45). The vaginal microbiota is also likely subject to the influence of an individual's innate and adaptive immunity, as well as practices including contraceptive method and sexual behaviors (46–48).

Longitudinal studies have found the vaginal microbiota to be relatively stable, with transient changes in composition tending to coincide with altered physiology (e.g., menstruation) or behavior (e.g., sexual activity, douching) (28, 49–51). In general, hormonal fluctuations during the menstrual cycle do not appear to dramatically change the vaginal microbiota, however hormones are certainly involved in its modification. The major hormonal shifts occurring at puberty significantly change the vaginal microbiota from mainly anaerobic bacteria to one dominated by lactobacilli (52). Conversely, at menopause the vaginal microbiota reverts to mainly anaerobic bacteria (53, 54). Unsurprisingly, estrogen is believed to be the main hormone responsible for these shifts, and a positive correlation between estradiol and lactobacilli concentration can be seen in post-menopausal women receiving estrogen-based hormone replacement therapy (53–55). Furthermore, the rise in hormones including estrogen during pregnancy stabilizes the vaginal microbiota; typically, the vaginal microbiota of a healthy pregnancy is *Lactobacillus* dominant (56, 57). The vaginal microbiota of pregnancy, and the correlation between dysbiosis and adverse reproductive outcomes has been reviewed elsewhere (58, 59). In addition to physiology affecting composition of the vaginal microbiota, sexual intercourse, use of antimicrobial agents, contraceptives, lubricants, vaginal douching, and other behaviors can also influence its composition [reviewed in (42)]. Indeed, it appears as though the microbiota fluctuates when exposed to a change in its *milieu*; one that enhances or diminishes the competitive advantage of certain vaginal microbes over others. For instance, both antibiotics and sexual intercourse modify host physiology or the vaginal microenvironment and are associated with a loss of vaginal lactobacilli (60). Similarly, menstrual blood at menses provides a change in substrate for microorganisms, and alters vaginal pH (61), consequently changing the microbiota composition [reviewed in (42)]. A recent study demonstrated that the vaginal (and uterine) microbiota changes with age. In the uterine cavity, bacterial alpha diversity (diversity within an individual's microbiota) decreases slightly with age, whereas the opposite effect (i.e., an increase in diversity) is observed in the vagina. In both locations, age and microbial diversity are correlated. The authors hypothesize that this phenomenon is a result of location: the vagina is not a closed environment, which increases its susceptibility to changing microbial composition, whereas the uterine cavity is “closed” which may confer greater stability of the microbiota in that location (62).

### The upper reproductive tract microbiotas

Moving up the FRT, the cervix, uterus, Fallopian tubes, and ovaries have distinct, and lower biomass microbiotas than the vagina (23, 63, 64). However, the existence of permanent microbiotas at these sites has been challenged. The upper reproductive tract was initially considered a sterile environment (65, 66), but studies show specific microbial patterns in the uterus, placenta, Fallopian tubes, and ovaries. The “sterile womb” assumption was challenged when it was reported that bacteria can ascend into the upper genital tract through the cervix (67).

The cervical microbiota plays a role in removing toxic compounds, strengthening the female genital tract epithelium, and regulating the immune system (68–70). Studies have also shown that the cervical microbiota is involved in carcinogenesis of the endocervix (71, 72). Tango et al. compared the cervical microbiota of healthy individuals and a high-grade cervical intraepithelial neoplasia and cervical cancer (CIN2/3-CC) group. They found that seven phyla (*Firmicutes, Actinobacteria, Bacteriodetes, Proteobacteria, Fusobacteria, Tenericutes*, and *Saccharibacteria*_TM7) were abundant in both groups, with the phylum *Saccharibacteria*_TM7 being less abundant in the CIN2/3-CC group compared to healthy controls (73). Another study investigated the association of the cervical microbiota with cervical cancer and reported that cervical pathology was associated with lower abundance of *Lactobacillus* and higher anaerobes. It has also been shown that in healthy women, there is a lower relative abundance of bacteria in the endometrial microbiota compared with the cervical microbiota (74–76).

For almost a century the uterus was believed to be sterile. This assumption was challenged by targeted PCR identification and culture-based technology at hysterectomy, and it was reported that the uterus has its own microbiota containing both *Lactobacillus* and non-*Lactobacillus* species (65, 77–79). More specifically, the uterine microbiota has an abundance of *Lactobacillus, Gardnerella, Prevotella* genera, and *Bacteroides* and shares Firmicutes and Actinobacteria with the vaginal microbiota. However, the endometrial microbiota has a lower relative abundance and a higher microbial diversity than the cervical and vaginal microbiotas (74, 75). The first evidence of bacterial colonization in the uterine cavity dates back to over 30 years ago where *Lactobacillus* spp., *Gardnerella vaginalis, Enterobacter* spp., and *Mycoplasma hominis* could be cultured from 25 to 30% of the samples obtained transcervically or after hysterectomy (80, 81). Later studies revealed the uterus contains bacterial taxa different from those found in vaginal samples (82) and that less bacterial DNA is detected in upper reproductive tract samples compared to vaginal samples, confirming that the microbiotas in the upper reproductive tract have a lower biomass compared to the vaginal microbiota (82). Chen et al. confirmed this and further demonstrated that the upper reproductive tract contains 10,000 less bacteria than vagina (23). This difference could be due to the cervical barrier inhibiting bacterial ascension from the vagina.

Pelzer et al. investigated the Fallopian tube microbiota in healthy women (7). It was reported that the Fallopian tubes have robust microbial communities dominated by members of the phyla Firmicutes, most notably *Lactobacillus* spp., *Staphylococcus* spp., and *Enterococcus* spp. Other common taxa were Pseudomonads (*Pseudomonas* spp. and *Burkholderia* spp.) and known genital tract anaerobes such as *Propionibacterium* spp. and *Prevotella* spp. (7). However, there are limitations and inconsistent findings between different studies. For example, Yu et al. reported that bacteria from Fallopian tubes samples were slightly above the negative controls, indicating the possibility of low-level bacteria in Fallopian tubes (8). Moreover, using 16S rRNA gene sequencing bacteria were detected in the Fallopian tubes, ovaries, uterus, and cervix from patients undergoing hysterectomy with bilateral salpingo-oophorectomy (83). It was reported that microbial communities of the Fallopian tubes, endometrium, myometrium, and ovaries were more varied in their compositions with the exception of *Lactobacillus*. Additional studies with larger sample sizes will be necessary to investigate and confirm the “core microbiota” associated with these sites (83). To the best of our knowledge, except for Miles et al. (83) there has been no other study investigating the ovarian microbiota in the healthy or diseased state.

Taken together, the upper reproductive tract is evidently not sterile, but there is limited research on the composition and function of the upper reproductive tract bacterial communities. This could be due to the complexity and invasive nature of collecting samples from the upper reproductive tract. Also, because most samples are collected during surgical and explorative procedures, most currently available data captures the upper reproductive tract microbiota composition in a diseased state. Therefore, whenever possible future studies should investigate the commensal bacteria and microorganisms of the reproductive tract in the absence of gynecologic disease.

### Biofilms

The presence of biofilms is another characterization of the vaginal microbiota. Biofilms are colonies of microorganisms covering a solid surface and they can be identified on the surface of vaginal epithelial cells. Other microorganisms, such as *Candida* spp. and *G. vaginalis*, exist in the cervicovaginal microbiome in addition to lactobacilli. Overgrowth of *Candida* spp. or *G. vaginalis* may lead to biofilm production, resulting in microbial dysbiosis and an increase in the risk of acquiring STIs. The inability of the immune system and antimicrobial agents like antibiotics to fully eliminate biofilms result in persistent infection and bacterial vaginosis (BV) has a higher relapse and recurrence rate as a result (84–86). BV, which will be discussed in greater detail below, contributes to more than 60% of all vulvovaginal infections which makes it the leading vaginal disorder in women of reproductive age (87). BV is the only clinical diagnosis related to the vaginal microbiota and is a polymicrobial condition characterized by low abundance of lactobacillus and overgrowth of anaerobes.

## Immune homeostasis with the female reproductive tract microbiotas

### Modulation of inflammation and immune homeostasis by commensal bacteria

Here, we will discuss the current state of knowledge surrounding the mechanisms by which epithelial and immune cells in the reproductive tract sense the microbiota. We will also discuss the complexities of these interactions and the limitations surrounding our current knowledge. Similar to other areas of the body harboring bacteria, the bacteria colonizing the FRT interact with their host. A bidirectional relationship exists between the bacteria and host wherein the bacteria appear to tune inflammation and immunity while the host immune system can modulate the microbiota. Native microbiotas within the FRT survive because they are tolerated by the host; the microbes are sensed by pattern recognition receptors (PRRs) that are present on both squamous epithelial cells lining the vagina and columnar cells lining the upper FRT, but an immune response is not directed against them. These receptors include dectin-1 receptor, toll-like receptors (TLRs), and nucleotide-binding oligomerization domain (NOD)-like receptors (88–92).

**Figure 2:**
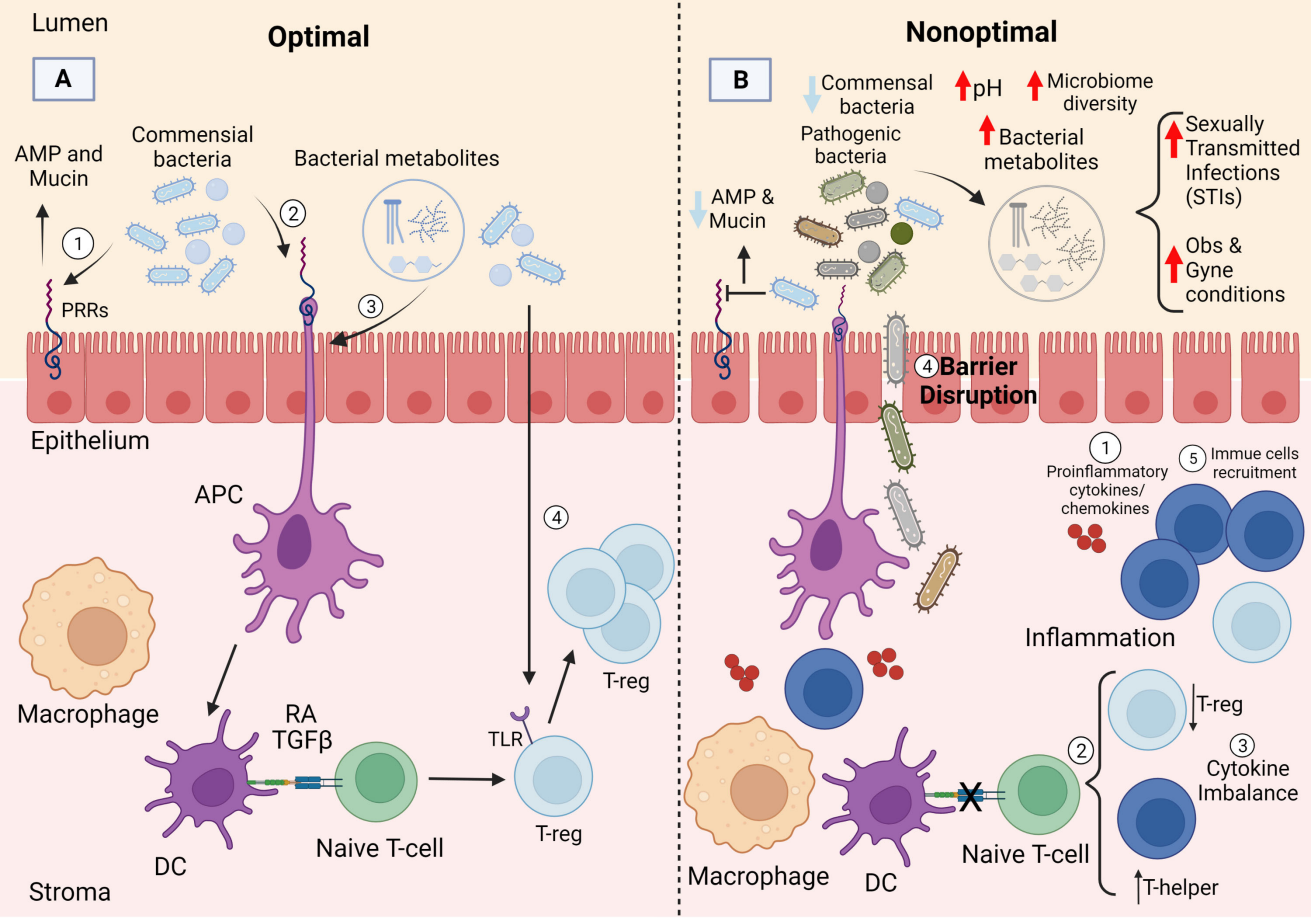
Potential mechanism of immune homeostasis with the female reproductive tract microbiotas. **(A)** Commensal bacteria likely maintain homeostasis by interacting with the epithelial cells *via* pattern recognition receptors (PRRs) and promoting induction of T-reg cells through different pathways: (1) commensal bacteria play an important role in preserving the epithelial barrier through antimicrobial peptide (AMP) and mucin production, (2&3) commensal bacteria or bacterial metabolites can induce antigen presenting cells, such as dendritic cells (DCs) that favor T-reg selection through production of retinoic acid (RA) and TGF-β, (4) commensals can also facilitate tolerance through signaling and triggering toll-like receptors (TLRs). **(B)** The loss of commensal bacteria can lead to increased microbial diversity and changes in immune and epithelial homeostasis likely through multiple mechanisms including: (1) production of pro-inflammatory cytokines and chemokines, (2) decrease in T-reg frequency and increase in T-helper cells; ultimately resulting in (3,4&5) cytokine imbalance, barrier disruption, and immune cell recruitment. Created with BioRender.com.

Epithelial cells in the FRT are colonized by commensal bacteria which play an important role in preserving an antimicrobial barrier against pathogens and thus help maintain an intact, stable, and protective epithelial barrier. This includes the secretion of antimicrobial peptides (AMPs) by commensal bacteria upon binding to the epithelial cells (93, 94). Additionally, commensal bacteria produce mucin which contributes to the stabilization of tight and adherens junctions in the FRT (65, 95). Moreover, commensal bacteria can modulate immune responses at the molecular level (Figure 2A). Although few mechanistic details (particularly in the upper FRT) are known about how commensal FRT bacteria interact with the host immune system, inferences can be gleaned by looking at the gut. For example, commensal bacteria play a critical role in the differentiation of CD4^+^ T cells (T helper cells) at the barrier sites and can induce both pro- and anti-inflammatory CD4^+^ T cell responses through different mechanisms in the gut (96). As first suggested by adoptive transfer experiments in rodents, CD4^+^ regulatory T (Treg) cells play an important anti-inflammatory role in maintaining tolerance to commensal bacteria in the gut (97) and preventing an inappropriate immune response to them (98–101). In the gut, studies suggest commensal bacteria maintain immune tolerance by triggering antigen specific Treg responses. For example, altered germ free mice introduced to Schaedler flora (a known community of 8 bacterial species) experienced an increased frequency of colonic Tregs as a result (102). *Clostridium* clusters XlVa and IV (103, 104), along with *Lactobacillus reuteri* (105), which are the major players in the gut microbiome, have also been shown to increase Treg cells frequency. Although little is known about immune tolerance to commensals in the FRT, it is important to note that *Lactobacillus reuteri* is a probiotic bacterium found in the urinary tract (106), that may modulate immune tolerance at this site. Fewer studies have focused on the role of Tregs in FRT. There is an inverse association between T-regs and pro-inflammatory cytokines in the endocervix; a higher frequency of endocervical Treg is associated with lower pro-inflammatory cytokines, such as IL-1β, IL-8, G-CSF, MIP-1β, Eotaxin, and IL-1RA (107). A higher endocervical T-regs concentration is also associated with lower CD4^+^ T cells which are required for human immunodeficiency virus (HIV) to establish itself in the mucosa, further suggesting an anti-inflammatory role for Tregs in FRT (107). Taken together, the are many knowledge gaps regarding the role of Tregs in FRT which need to be addressed in future studies. Moreover, antigen presenting cells (APCs) [such as dendritic cells (DCs)] favor Treg selection in the gut because they produce higher levels of retinal dehydrogenase which produces the vitamin A metabolite retinoic acid (RA) (108–110). RA contributes to homeostasis of the gut microbiota through inhibition of cytokine production by effector cells (111) and promoting Treg cell selection (112). Dendritic cells can produce transforming growth factor β (TGF-β) which can further contribute to Treg selection in combination with RA (113). O'Shea and Paul reported that T cells contribute to homeostasis in the gut by promoting Treg cells in the presence of TGF-β, RA, and IL-2 (114). Even though unlike gut, the role of APCs in Treg selection in FRT is understudied, APCs have been reported to also play an important role in directing T cell responses in FRT (115). Duluc et al. for the first time demonstrated that human vaginal mucosa harbors four major myeloid-originated APC subsets (LCs, CD14^**−**^ LP-DCs, CD14^+^ LP-DCs, and Mϕ) which direct T cells to orchestrate the vaginal immune responses (115). Moreover, Tan et al. reported that mice receiving all-*trans*-retinoic acid were more resistant to vaginal infections due to a stronger T cell recall response *in situ*, suggesting the role of RA in T cell selection and FRT homeostasis (116). With respect to FRT homeostasis, TGF-β has been mostly studied in relation to fetal-maternal immune tolerance. TGF-β has been shown to assist estradiol in inhibition of vaginal antigen presentation and orchestrating immune response against sperms (117) along with increasing the vaginal Tregs to help reduce fetal loss (118). TGF-β also plays a role in immunosuppression at different menstrual cycle stages (119, 120). Another plausible pathway for Treg selection is through microbiota itself. Commensal bacteria have been reported to facilitate tolerance through signaling and triggering TLRs in both the gut and FRT (121). Fazeli et al. reported that TLR4 which is present in endocervix, endometrium, and uterine tubes, may play an important role in immune tolerance in the lower parts of the FRT (122). Moreover, different studies have shown that vaginal and uterine Treg cells play an integral role in maintaining a healthy microbiota during pregnancy and homeostasis (123, 124) (Figure 2A).

Interplay between the FRT microbiotas and cells of the immune system exists to prevent infections with other potentially harmful bacteria and to create an immune-tolerant environment (18). When vaginal bacterial homeostasis is disturbed (as in both symptomatic and asymptomatic BV), epithelial cells become damaged and apoptotic, an effect mediated by caspase-3 activation (125, 126). Thus, the microbiota is critical in maintaining tissue integrity and homeostasis. The female sex hormones estrogen and progesterone also play an essential role in the interplay between the FRT microbiotas and immune system by inducing production of pro-inflammatory cytokines, such as IL-6 and IL-8, and anti-microbial peptides, such as alpha- and beta-defensin, by vaginal epithelial cells to help prevent infection (127). Steroid hormones, such as estrogens and progesterone, modify both adaptive and innate immunity (128–130). Estrogens regulate the expression of genes involved in proliferation, reproductive functions, and cell survival by binding to estrogen receptor alpha (ERα) or ERβ, transcription factors (TFs) of nuclear receptor (NR) superfamily (131, 132). Estrogens have both anti- and pro-inflammatory effects depending on the context and target gene (133, 134). On the other hand, progesterone which can act of DCs, *T*-cells, and macrophages, exert its anti-inflammatory effects by decreasing leukocyte activation and producing pro-inflammatory mediators (130). Additionally, sex hormones can modify bacteria; for example, estrogen was shown to increase the survival and growth of Gram-negative bacteria and estradiol can enhance the virulent mucoid biofilm phenotype of *Pseudomonas aeruginosa* (135). Additionally, an *in vitro* study showed that estrogen can increase the growth of *Escherichia coli* (136) which aligns with *E. coli* causing urinary tract infections (UTIs), one of the most commons infections in women (137).

Cytokine production is an important part of the host immune response and essential for protective immunity. For instance, the reproductive tract is capable of increasing IL-1β concentration followed by IL-8 in response to the presence of pathogens and plays an important role in activating both adaptive and innate immune response against BV-associated bacteria (138). Moreover, TLRs on mucosal cells can bind and identify a broad range of bacterial pathogen-associated molecular patterns (PAMPs) and initiate a signaling cascades aimed at clearing the infection if required (139, 140). However, sustained production of cytokines can cause epithelial barrier damage and induce T cell infiltration into the genital mucosa, which can be detrimental to the FRT (141), and lead to increased infections.

### Potential mechanism of dysbiosis in FRT

During homeostasis, a balance exists between the FRT bacteria and the immune system; one that maintains immune quiescence. Although understudied, it appears as though Tregs are involved in tolerance of the cervical and vaginal microbiotas (142). However, it seems this balance can be tilted toward inflammation under the influence of certain factors (e.g., stress, disease, infection, pathology, antibiotic therapy, sexual practices, lifestyle choices, etc.), perhaps due to their direct effect on bacteria or their indirect effect on the microenvironment supporting the bacteria. Moreover, it has been shown that some stresses such as poor housing conditions, poor nutrition, low income, and interpersonal conflicts increase the risk of BV (143, 144). For example, it was found that a 5-unit-increase in Cohen's Perceived Stress Scale was associated with higher risk of acquiring BV, suggesting that stress can change the composition of the vaginal microbiota (145). Moreover, maternal stress can change the abundance of vaginal *Lactobacillus* and proteins related to vaginal immunity during pregnancy (146).

Loss of *Lactobacillus* dominance leads to increased microbial diversity and changes in immune and epithelial homeostasis in the vagina through multiple mechanisms, such as production of pro-inflammatory cytokines and chemokines, immune cell recruitment, and reduction in the viscosity of the cervicovaginal fluid (147, 148) (Figure 2B). Perturbances to the bacteria, immune system, or local environment may result in microbial dysbiosis, defined as an atypical microbiota composition. However, the direction of initiation of dysbiosis is largely unknown (e.g., whether overgrowth or a change in FRT bacteria due to a perturbance results in dysbiosis, or whether an immune response alters the native microbiota and results in dysbiosis), although it seems reasonable to expect both situations are possible. Nevertheless, many studies discuss an association between inflammatory states and bacterial dysbiosis. For example, alterations in the vaginal microbiota inhibit chemokine secretion and chemotaxis and increase pro-inflammatory cytokines [reviewed in (18)]. Additionally, an increase in anaerobic bacteria and loss of *Lactobacillus* dominance increases bacterial diversity and can lead to dysbiosis of the vaginal microbiota. Furthermore, diversity of the vaginal microbiota has been linked to a compromised genital epithelial barrier, high levels of mucosal inflammation, and increased risk of STIs such as (HIV) (149, 150). Moreover, it has been shown that unlike the gut microbiome (151, 152) the short chain fatty acids (SCFAs) in the vagina contribute to the pro-inflammatory environment and less significantly to antimicrobial activities (153, 154). The concentration of SCFAs increase during vaginal dysbiosis which enhances TLR2 and TLR7 ligand-induced production of IL-8 and TNF-α in a dose-dependent manner. SCFAs also mediate pro-inflammatory cytokine production partly by generation of reactive oxygen species (ROS) (155).

Although much of our knowledge on the mechanisms governing immune tolerance to the native microbiota comes from the gut literature, a few studies are beginning to provide support for a role for the female reproductive microbiotas in maintaining immune tolerance, and tuning inflammation (Figure 2).

## Microbial dysbiosis, inflammation and gynecologic conditions

### Microbial dysbiosis and inflammation

#### Bacterial vaginosis

Vaginal dysbiosis has been defined as a perturbance to the resident microbiota in the vaginal tract (156) or as a non-*Lactobacillus* dominant vaginal microbiota (150). Regardless of the precise definition, the literature agrees that dysbiosis is a departure from homeostasis between the FRT bacteria and the immune system. Bacterial vaginosis (BV), a condition characterized by non-optimal vaginal microbiota, is common among reproductive age women (157). A shift in vaginal bacteria toward a non-optimal state can increase the risk of BV, UTIs, or STIs such as HIV (42, 62). There has been a recent shift to use the term “non-optimal” over “dysbiotic” in the context of BV, as the latter may inadvertently stigmatize individuals with asymptomatic BV and low genital inflammation, whose microbiota represents their baseline or “healthy” state (157). The available diagnostic criteria for BV are the Amsel criteria and Nugent score (158). The Amsel criteria is a clinical assessment based on vaginal discharge, vaginal pH > 4.5, the presence of clue cells in a wet mount, and a “whiff test” (amine odor when vaginal secretions are treated with potassium hydroxide solution). To diagnose BV, 3 out of 4 criteria must be present (159). The Nugent scoring system is a laboratory-based method that evaluates Gram staining and the concentration of lactobacilli and other bacterial morphotypes in a vaginal smear (160). This system is considered the diagnostic gold standard, however both diagnostic methods are equally reliable, therefore the Amsel criteria are an efficient and cost-effective diagnostic alternative when lab equipment is not readily available (158). Interestingly, the extent of vaginal dysbiosis is usually positively correlated with Nugent score and vaginal pH (150). However, we and others report this is not always the case (28, 48). In our previous study 63% of sex workers with a diverse vaginal microbiota classified as CST-IV by 16S rRNA gene sequencing had low Nugent scores and were not symptomatic for BV (48). These findings call the current concept of a *Lactobacillus* dominant vaginal microbiota as “healthy” into question. Not all individuals with BV are symptomatic (157) and evidently microbial diversity can exist in some people in the absence of clinical symptoms or a Nugent score indicative of BV. As the aforementioned study did not exclude the possibility that subjects with a polymicrobial vaginal microbiota in the absence of BV had genital inflammation, they would be colonized by a non-optimal vaginal microbiota as defined by McKinnon et al. (157). An interesting consideration for future research would be to assess inflammation in women who have a polymicrobial vaginal microbiota in the absence of BV by Nugent score. We propose that perhaps every individual has a different threshold of bacterial tolerance, and only once this threshold is reached will an immune response be elicited.

#### The inflammatory signature associated with microbial dysbiosis of the FRT

In optimal and non-optimal conditions alike, cytokines regulate the vaginal microbiota (161). When any pathogen or “foreign invader” reaches the vaginal mucosa, the mucus, epithelial cells, and underlying immune cells offer the first line of resistance poised to counter the threat. However, vaginal dysbiosis can elicit an immune response (162), induce inflammation, and compromise this first line of defense (163). Genital inflammation also ensues as the host fights the pathogen or STI (164). Antigen presenting cells, CD4+ T cells, and epithelial cells mediate FRT inflammation (165). Having an intermediate vaginal microbiota, defined as a Nugent score between 4 and 6, or BV (Nugent score ≥ 7) is associated with an increased rate of incident *Neisseria gonorrhoeae, Trichomonas vaginalis, Chlamydia trachomatis* or human papilloma virus (HPV) infection (164). The main mechanism by which this occurs is that BV induces chronic inflammation, which consequently disrupts the epithelial barrier (164), granting the infections easier access to underlying tissues and cells. When microbes activate PRRs expressed on epithelial cells lining the FRT (specifically the squamous epithelial cells that comprise the vaginal lining and columnar cells of the upper FRT), they initiate a signaling cascade of cytokines and chemokines. Cytokines including IL-1β, TNF-α, IL-6, and IL-8 are secreted by the epithelial cells lining the vaginal tract, which recruit or activate cells of the innate and adaptive immune systems, including but not limited to macrophages and cytotoxic (CD8+) T cells, respectively (17).

Many authors have extensively studied the inflammatory signature of women with a polymicrobial vaginal microbiota. Higher bacterial diversity is associated with greater inflammatory cytokine concentrations (164), and one of the strongest independent predictors of genital inflammation in a recent study was a vaginal microbiota subtype that predominantly included women with high Nugent scores (i.e., increased microbial diversity) (166). Indeed, BV is characterized by a proinflammatory vaginal mucosa (25). Associations between BV and individual inflammatory mediators in cervicovaginal samples are inconsistent, however most studies report that cervicovaginal secretory leukocyte protein inhibitor (SLPI), an antimicrobial peptide, is decreased while IL-1β is increased [reviewed in (92)]. IL-1β, IL-8, and IL-6 differentially regulate biofilm growth and production by vaginal microbiota. Specifically, IL-1β and IL-8 promote *S. aureus* and *E. coli* to grow and produce biofilms (161). The presence of *Dialister micraerophilus, Eggerthella* type 1, and *Mycoplasma hominis* in vaginal swabs is associated with elevated vaginal TNF-α levels. Detection of the aforementioned bacterial species with the addition of *Parvimonas* type 2 is associated with elevated vaginal IL-1β concentrations (167). IL-1β can direct the activation of naïve cord blood CD4+ T cells to a pro-inflammatory phenotype, which induces secretion of other proinflammatory cytokines *in vitro* (168). Women with a high diversity cervicovaginal microbial community such as CST-IV, and an associated elevation in genital inflammation, have high numbers of HIV-infectable CD4+ T cells in the cervix (169). Anahtar et al. demonstrated this relationship between pro-inflammatory cytokine levels and high bacterial diversity (i.e., low *Lactobacillus* concentrations) of the cervicovaginal microbiota in asymptomatic HIV-negative women from South Africa. Compared to CST-III women, CST-IV women are four times as likely to have high levels of pro-inflammatory cytokines in the genital tract. A CST-IV microbiota is a stronger predictor of inflammation than having an STI or BV (169). CST-IV and herpes simplex virus (HSV)-2 infection enhance HIV susceptibility (170). Furthermore, IL-8, IL-1β, and IL-1α are elicited at higher levels by *Prevotella amnii, Mobiluncus mulieris, Sneatha sanguinegens* and *S. amnii* compared to *L. crispatus* (169). Increased mucin 5B and 5AC, increased proteolytic activity, and elevated proinflammatory cytokines are associated with increasing bacterial diversity of cervicovaginal samples (171). High diversity community state types and the presence of *Gardnerella vaginalis* and *Prevotella bivia* are associated with cervicovaginal inflammatory cytokines (170). Campisciano et al. were the first to characterize the clinical impact of cytokine modulation of vaginal dysbiosis. A distinct pattern of inflammatory mediators—IL-1β, IL-8, MIG, MIP1-α, and RANTES—distinguished the severity of vaginal dysbiosis (162). To further illustrate the link between polymicrobial bacterial communities and inflammation in the lower FRT, treatment of BV with the antibiotic metronidazole in HIV-infected women decreases IL-1β, IL-8 and RANTES in cervical samples (172). Elevated IL-5 and IL-13, two Th-2 secreted cytokines, are associated with depleted *Lactobacillus* spp*., G. vaginalis*, and *Ureaplasma* spp. (162). Th-2 cells are a subset of CD4+ T-cells; Th-2 cytokines create an anti-inflammatory response, which balances the complementary Th-1 mediated proinflammatory response (173). The results of the Campisciano et al. study suggest that Th-2 activation may have a role in restoring eubiosis in the vagina, and the authors suggest IL-5 and IL-13 could be explored as indirect markers of vaginal dysbiosis (162). A recent study established a temporal relationship between dysbiosis and cervical immunity (156). By studying a longitudinal cohort of HIV-negative women (22% of which acquired HIV during the study), the authors were able to report patterns of cervical immunity that preceded and predicted vaginal dysbiosis [which they defined as candidiasis, BV, or an intermediate Nugent score (4–6)]. This pattern included a proinflammatory state that consisted of upregulated IL-1 signaling, and downregulation of SLPI. This study appears to be the first to provide evidence that changes in genital tract immunity can precede cervicovaginal infection or an altered vaginal microbiota (156). Taken together, the literature appears to suggest that vaginal homeostasis can be disrupted by two different mechanisms: first, a shift toward bacterial diversity can induce vaginal inflammation, and second, that local immunity is altered in some manner, and the microbiota change in response to environmental alteration.

#### Microbial dysbiosis and inflammation in the upper FRT

Much of our understanding of the link between microbial dysbiosis and inflammation in the FRT is derived from studies with vaginal and cervicovaginal samples, and thus a large gap in the literature remains with respect to uterine, Fallopian, and ovarian dysbiosis as they relate to inflammation. While the microbiota of the upper FRT remains largely understudied, limited data is available on the balance between the microbiota and immunity of FRT structures distal to the vagina. *Ureaplasma parvum*, a bacterium associated with pregnancy complications such as preterm birth, can colonize cervical epithelial and stromal cells, and weakly induce inflammation. *U. parvum* increases the expression matrix metalloprotein (MMP)-9 in endocervical epithelial cells and initiates the release of pro-inflammatory cytokines IL-6 and IL-8 in both epithelial cells of the endo- and ectocervix and cervical stromal cells (174). In this study, the inflammatory effects of *U. parvum* do not seem to account for preterm birth (174) but may be one part of a larger interaction between the epithelium, bacteria, and inflammatory mediators. These findings provide insight into the intimate relationship between bacteria and the innate immune response at the FRT epithelial barrier beyond the vagina. Furthermore, in a seminal experimental animal study Wang et al. described microbial translocation from the vagina to uterus and the ensuing effects on uterine health (62). The authors used a rat model to assess the possibility that vaginal bacteria can ascend the cervix and transplant in the uterus, and to explore the impact of dysbiosis on the uterine microenvironment. Vaginal microbiota transplant from women with chronic endometritis, BV, or healthy controls was performed *via* vaginal lavage (62). Chronic endometritis—distinct from endometriosis—is a benign, generally asymptomatic gynecological condition caused by infection that is frequently associated with poor outcomes during assisted reproduction (175). Rats that received a transplant from women with chronic endometritis had greater uterine inflammation, quantified by elevated mRNA levels and concentrations of TNF-α, IL-1β, and CD38. This demonstrates that the vaginal microbiota can induce uterine inflammation, possibly following translocation *via* the cervix. Further animal experiments in the same study revealed that *P. bivia* or *Clostridium perfringens* transplanted into the vagina can translocate to the uterus, initiate inflammation, and promote the formation of endometritis-like lesions (62). Inflammation and dysbiosis within the FRT are often studied at single sites, but this valuable research reminds us that nothing exists in isolation. These findings allow us to extend our understanding of FRT dysbiosis, revealing that the spread of bacteria between body sites may maintain or disturb homeostasis (62). Future studies that examine the inflammatory effects and crosstalk between bacteria at multiple sites in the FRT will continue to shed light on complicated states of FRT dysbiosis and the implications for reproductive health and disease.

The current literature linking inflammation and vaginal dysbiosis presents comprehensive results derived from robust sequencing and transcriptomic techniques and implies there may be clinical applications to improve reproductive outcomes and diagnosis of vaginal infections. However, it is important to consider the populations from which study results were drawn because of the impact of host genetics, physiology, or behavior on the vaginal microbiota. For example, several studies examine the vaginal microbiota in African women (156, 165, 167, 169–171, 176) due to the geographic burden of dysbiosis and HIV infection in this region (42, 150, 165). One study evaluated the association between vaginal microbiota composition and ethnicity in a sample of women representative of the six dominant ethnic groups in Amsterdam, Netherlands (177). Kumar et al. analyzed the vaginal cytokine composition and microbiota of Asian women in association with delivery outcomes (178) and Campisciano et al. studied the pathogenesis of vaginal dysbiosis in a Caucasian population (162). It has been established that community state frequency differs across ethnic backgrounds, as previously discussed. Therefore, when evaluating the cellular consequences of FRT dysbiosis and extrapolating the clinical relevance of these findings, it is essential to consider the populations in which these results were obtained. The concept of health vs. dysbiosis in the FRT appears to be more complicated than a binary; rather, microbiota composition exists on a spectrum that may be intrinsically different across diverse people because of genetics, physiology, behavior, and environment. Future research should aim not only to capture this complex diversity, but to improve the utility of diagnostic tools and treatments for FRT dysbiosis by validating or tailoring their use in diverse populations.

### Female reproductive tract microbiotas and gynecologic conditions

A balance between the resident microbiota and the immune system appears to be necessary to support reproductive health, and an altered microbial balance and inflammation can have negative effects. As mentioned above, the largest body of literature linking the FRT microbiotas to gynecological conditions is around STIs; in particular, dysbiosis of the FRT microbiota is associated with an increased risk of HIV acquisition (18, 156, 164, 167, 169, 171, 179). Anahtar et al. proposed the mechanism behind this association was that epithelial and antigen-presenting cells sense cervicovaginal bacteria and activate inflammation *via* the NF-kB and toll-like receptor pathways (169). Secreted chemokines recruit activated CCR5+ CD4+ T cells (which are the target cells for HIV to infect) to the site of inflammation. The authors hypothesized that TNF-α and IFN-α may disrupt tight junctions of the endocervical columnar epithelial barrier, which would make it easier for HIV to access underlying target cells, and dually increase the likelihood of infection (169). This is supported by primary *in vitro* research demonstrating that TNF-α secreted following HIV-1 exposure disrupts epithelial barrier function (180); IFN-γ also increases tight junction permeability in cultured human intestinal epithelial cell monolayers (181).

Robertson et al. argue that the cytokines present during the pre- and peri-implantation period are essential for fetal development and pregnancy outcome (182). Granulocyte-macrophage colony-stimulating factor (GM-CSF) is essential for blastocyst development, whereas the pro-inflammatory cytokines TNF-α and IFN-γ have inhibitory effects on blastocyst development. A symphony of cytokines orchestrates the window for embryo implantation, including host immune tolerance of the embryo. Experiments in a mouse model have demonstrated that systemic exposure to low-dose lipopolysaccharide (LPS; an endotoxin expressed by Gram-negative bacteria) during the implantation period reduces embryo cell number, embryo viability, and oviduct pro-inflammatory cytokine expression (182). Like this delicate cytokine balance, the presence of an “optimal” microbiota in the FRT also seems important in the maintenance of fertility, and FRT dysbiosis is linked to pregnancy-related adverse outcomes including preterm birth, spontaneous abortion, and infertility [reviewed in (10)]. Women with both tubal infertility and *Chlamydia trachomatis* infection have an increased abundance of vaginal *L. iners* relative to *L. crispatus* and an overall decrease in *Lactobacillus, Atopobium, Streptococcus, Bifidobacterium*, and *Enterobacter* genera (183). During the second trimester of pregnancy, the vaginal microbiota is more diverse in pregnant women living with HIV than HIV-uninfected pregnant women. There is a higher prevalence of *L. iners* dominant vaginal microbiota and lower prevalence of *L. crispatus* dominance among pregnant HIV-infected women (184). In general, *L. crispatus* dominance is more beneficial to vaginal health over *L. iners* (163). Increased bacterial diversity and the presence of anaerobic bacteria is associated with cervicovaginal inflammation in pregnancy, which may contribute to preterm birth which is frequently experienced by pregnant HIV-infected women (184). These studies shed light on the interconnectedness of FRT infection, microbial balance, and reproductive outcomes.

Beyond STIs, the FRT microbiotas also seem to be involved in conditions including infertility; endometriosis; endometrial, cervical, and ovarian cancer; and polycystic ovary syndrome (PCOS). Although the mechanism(s) by which bacteria might be involved in these gynecological conditions remain speculative at present, it may in part be *via* the intimate link we are beginning to discover between the FRT bacteria and the host immune system. As inflammation is a central aspect that unites these gynecologic conditions, we will now explore the few studies reporting perturbations in FRT microbiotas as they relate to gynecologic conditions.

#### Endometrial microbiota and infertility

Although most studies focus on the vaginal microbiota (with the traditional definition of vaginal health/optimal bacteria being *Lactobacillus* dominant), there are some studies reporting associations between other FRT microbiotas and gynecological conditions, however, there is no consensus on what constitutes an “optimal” microbiota at these other anatomical locations. As previously discussed, this is in part because most studies on FRT microbiotas (other than the vaginal microbiota) lack a “healthy” control group but rather include women with gynecological conditions, and there may be an increased risk of sample contamination when upper FRT microbiotas are collected because this is typically a transcervical procedure (185). However, Moreno et al. found that in infertile women seeking *in vitro* fertilization (IVF), a non-*Lactobacillus* dominant endometrial microbiota during the receptive phase of the cycle was associated with significantly reduced implantation rates, pregnancy, ongoing pregnancy, and live birth compared to a *Lactobacillus* dominant microbiota (63). A lower median percentage of endometrial *Lactobacillus* was reported in women undergoing IVF compared to healthy volunteers and non-IVF patients (186). Together these studies suggest that *Lactobacillus* dominant endometrial microbiota is favorable for embryo implantation (63, 186). Non-*Lactobacillus* dominant microbiota may trigger an inflammatory response that hinders embryo implantation (63). Additionally, a recent study points to the potential role of vaginal dysbiosis in primary ovarian failure (POF) (187), i.e., failure of ovarian function in women under the age of 40. Women with POF have significantly higher vaginal microbial diversity compared to healthy controls. Ten bacterial genera, including *Gardnerella, Prevotella*, and *Bacteroides* are abundant in the POF vaginal microbiota (187).

A recent prospective study by Moreno et al. greatly enriches our understanding of the relationship between reproductive tract microbiota and reproductive success, as the authors demonstrated the endometrial microbiota before embryo transfer during IVF is associated with reproductive outcomes (188). Specifically, patients with live births were more likely to have a *Lactobacillus* rich microbiota. An endometrial microbiota profile of *Atopobium, Bifidobacterium, Chryseobacterium, Gardnerella, Haemophilus, Klebsiella, Neisseria, Staphylococcous*, and *Streptococcus* was associated with poor IVF outcomes defined as biochemical pregnancy, no pregnancy or clinical miscarriage (188). However, others report that IVF failure is associated with enriched uterine *Lactobacillus*, suggesting that bacterial translocation from the vagina to endometrium negatively impacts embryo implantation (189). The disparity across studies highlights that the “desirable” endometrial microbiota composition for successful pregnancy has not yet been confirmed, but regardless, its composition seems to influence IVF success. Indeed, a recent systematic review concluded that the endometrial, cervical, and vaginal microbiotas may play a role in fertility and assisted reproduction outcomes, however a definitive relationship remains unclear due to an overall lack and inconsistency in the available data (190). Our understanding of the endometrial microbiota in pregnancy is further complicated by a lack of knowledge of its composition in fertile women who typically do not seek reproductive technologies and are therefore largely underrepresented in these studies. A procedure characterizing the endometrial microbiota from endometrial fluid or biopsy may be a promising future biomarker to predict reproductive outcomes prior to assisted reproduction (188). While much of the existing literature emphasizes the role of the FRT microbiotas in pregnancy [as reviewed elsewhere in (16, 17)] and STIs [reviewed in (18, 19)] there is evidence supporting the contribution of FRT microbiota to infertility.

#### Endometriosis

Endometriosis is a chronic systemic inflammatory condition marked by the presence of endometrial-like tissue in ectopic locations. This debilitating condition affects up to 1 in 10 people assigned female at birth (191). Endometriotic lesions are estrogen-dependent (192) and growth factors, inflammasomes, and pro-inflammatory cytokines contribute to an inflammatory peritoneal microenvironment which promotes the growth of endometriotic lesions (193). To further complicate our understanding of the pathophysiology of endometriosis, there is some evidence supporting a bidirectional relationship between endometriosis and the human microbiomes (194).

Endometriosis is associated with dysbiosis of the gut and reproductive (vaginal, cervical, and endometrial) microbiotas [reviewed in (195)]. Increased abundance of Proteobacteria, *Streptococcus* spp., *Enterobacteriaceae*, and *E. coli* at different microbiome locations seems to be associated with endometriosis (194). Several studies profiled the FRT microbiotas in women with endometriosis and demonstrated differences vs. control groups. For example, stage III endometriosis is associated with increased bacterial richness and phylogenetic diversity in urogenital samples (196). Compared to healthy controls, women with stage III-IV endometriosis have increased abundance of a range of pathogenic bacterial species (*Gardnerella, Shigella, Streptococcus, Escherichia*, and *Ureaplasma)* in the cervical microbiota. Furthermore, women with this stage of disease lack *Atopobium* in the cervical and vaginal microbiota (197). In women with endometriosis, the endometrial microbiota is enriched with the *Actinobacteria* phylum, *Oxalobacteraceae* and *Streptococcaceae* families, and *Tepidomonas* genus compared to symptomatic controls (6). Even ectopic endometriosis lesions display distinct bacterial compositions; deep endometriotic lesions have diminished *Lactobacillus* concentrations and elevated *Alishwanella, Enterococcus* and *Pseudomonas* (198), suggesting there is some incompletely understood link between bacteria and endometriosis.

Furthermore, there is evidence from experimental animal models that the microbiota beyond the reproductive tract is influenced by endometriosis. Mice with surgically induced endometriosis have lower *Firmicutes* and higher *Bacteroidetes* in the gut compared to control mice (193). When treated with broad-spectrum antibiotics, mice with induced endometriosis had significantly smaller lesions and reduced cell proliferation compared to vehicle-treated mice. This antibiotic regimen resulted in a reduced inflammatory response, as measured by lower IL-1β, TGF-β1, TNF-α, and IL-6 in peritoneal fluid and fewer macrophages present in endometriotic lesions. Metronidazole, an antibiotic that targets *Bacteroidetes*, reduced lesion growth. The authors suggested that gastrointestinal bacteria promote inflammation, which fuels endometriosis (193). Salliss et al. reviewed 6 animal studies and 28 clinical studies to conclude that endometriosis is associated with enriched bacterial diversity of the genital and gut microbiotas; in human studies, endometriosis and infertility are usually associated with *Lactobacillus* depletion and the presence of BV-associated bacteria in the cervicovaginal microbiome (199). Still, there is not a single microbiota profile characteristic of endometriosis. It remains unclear whether endometriosis contributes to dysbiosis in the FRT, or whether dysbiosis contributes to the pathophysiology of endometriosis (195). Nevertheless, epidemiological studies also support a link between bacteria and endometriosis; two large population-based studies found a 2-3X increased risk of having endometriosis in women with previous lower genital tract infections or pelvic inflammatory disease (ascension of pathogenic bacteria from vagina to upper FRT) (200, 201). Returning to the complex link between dysbiosis and inflammation in gynecological disease, it has been suggested that fertility outcomes in conditions like endometriosis and PCOS are partially mediated by dysregulated cytokines in the FRT (182), perhaps a result of microbial dysbiosis.

#### Endometrial cancer and endometrial polyps

Aside from infertility and endometriosis, microbial dysbiosis may contribute to other gynecological conditions including endometrial cancer and endometrial polyps. Endometrial cancer is the most common malignant gynecologic condition in the United States, with both incidence and death rates increasing annually (202). The pathogenesis of endometrial cancer remains poorly understood, but current research points to a putative role of FRT microbiota in its origin or progression. Across the vaginal, cervical, and endometrial microbiota, women with endometrial cancer have enriched Firmicutes, Spirochaetes, Actinobacteria, Bacteroidetes, and Proteobacteria. The presence of *A. vaginae* and *Porphyromonas*, in addition to elevated vaginal pH, is significantly correlated with endometrial cancer (5). Furthermore, mRNA expression of IL-6, IL-8, and IL-17 in the endometrial microenvironment is significantly different between women with endometrial cancer vs. benign uterine lesions. *Micrococcus* abundance in the endometrial microbiota is positively correlated with IL-6 and IL-17 mRNA, which supports a plausible link between the endometrial microbiota and inflammation in women with endometrial cancer (203). The role of reproductive and gut microbiota dysbiosis and inflammation in endometrial cancer is reviewed in Boutriq et al. (204).

Endometrial polyps (EPs) are benign lesions that result from overgrowth of the endometrium (205). EPs are often identified upon examination in patients experiencing abnormal vaginal bleeding or infertility (4). At the phylum level, “healthy” women, and women with EP (with or without chronic endometritis) have a uterine environment dominated by Proteobacteria, Firmicutes and Actinobacteria. However, the relative abundance varies across groups: compared to healthy controls, women with EP have significantly elevated Firmicutes and depleted Proteobacteria. Interestingly, uterine *Lactobacillus* and *Bifidobacterium* are elevated in women with EP (4). Prior research demonstrates that these species inhibit apoptosis and upregulate cell proliferation, suggesting that *Bifidobacterium* and *Lactobacillus* might be involved in the etiology of EP (4). Finally, the *Enterobacter* genus is diminished in the uterine microbiota of EP patients, which may contribute to the characteristic endometrial overgrowth (4). Several studies have characterized the composition of the endometrial microbiota and demonstrated that a bacterial signal in the uterus exists above what might be considered contamination (185). Additionally, studies further demonstrate the association of endometrial microbial diversity with a variety of diseases of the endometrium, as well as adverse reproductive outcomes (4, 6, 63, 188, 203).

#### Cervical cancer

Cervical cancer, the fourth most common type of cancer among women around the globe (206), is caused by persistent HPV infection and compounded by other factors including BV and STIs (207). The local immune, microbial, and metabolic signatures in the FRT may favor HPV persistence, putting the individual at greater risk for neoplastic disease (163). The presence of TGF-β1 and IL-10, two immunosuppressive cytokines, in the cervical cancer microenvironment enables HPV infection persistence (207). A pilot study suggests the cervical microbiota may modify local cytokine expression during cervical cancer development (207). The authors assessed microbial diversity and cytokine expression in cervical specimens from women with non-cervical lesions (HPV-positive or negative), squamous intraepithelial lesions, or cervical cancer using a classification with eight community state types to categorize microbial diversity. CST-VII samples, composed mostly of cervical cancer cases, were dominated by *Fusobacterium spp*. and had higher median levels of IL-4 and TGF-β1 (207). It was found that the expression of common cancer biomarkers in cervicovaginal lavage is correlated with genital inflammation and vaginal *Lactobacillus* abundance (208). Two out of three patient clusters identified in this study (cancer-associated and high diversity/inflammation groups) lacked *Lactobacillus* dominance, had high vaginal pH and genital inflammation, in addition to elevated local expression of cancer biomarkers. These features are associated with HPV infection persistence and cervical cancer. The biomarkers investigated in this study, which distinguished patients with increased genital inflammation and vaginal dysbiosis vs. “healthy” women, could be applied to predict HPV persistence, and consequently cervical cancer risk (208). There is an increased abundance of Actinobacteria, Proteobacteria, Bacteroidetes, and Fusobacteria in the cervicovaginal microbiota of women with persistent HPV infection (209). Cervical secretions from women with HPV persistence (and therefore cervicovaginal dysbiosis) have upregulated IL-6, TNF-α and immunosuppressive cells compared to women with transient or an absence of infection (209). Overall, several studies support a link between a polymicrobial vaginal microbiota with HPV persistence and consequently invasive cervical cancer development (210). The increasing body of literature supporting the involvement of the FRT microbiota and local inflammation in cervical cancer and other gynecologic malignancies is reviewed in more detail elsewhere (10, 163, 210–213).

#### Ovarian cancer and polycystic ovary syndrome

Moving up the FRT, there is evidence linking vaginal dysbiosis to ovarian cancer and PCOS. As discussed, there are barriers to studying the bacterial composition of the upper FRT although bacteria have been identified in both cancerous and non-cancerous ovarian tissue (214). Oncobiosis (i.e., dysbiosis in a cancerous state) has been identified in ovarian cancer cases in vaginal, cervicovaginal, upper genital tract, intra-tumoral, and ovarian samples, in addition to peritoneal fluid, serum, and fecal matter (215). Vaginal *Lactobacillus* dominance is a protective factor against ovarian cancer (216). Meta-analysis established that pelvic inflammatory disease is a risk factor for epithelial ovarian cancer (217); organisms including *C. trachomatis* and *N. gonorrhea* can lead to the establishment of this disease (216), further supporting a possible link between the trifecta of bacteria, inflammation, and gynecologic disease. Inflammation itself drives oncogenesis, which seems to be the case in the pathogenesis of ovarian cancer (215). The specifics of oncobiosis in ovarian cancer include decreased *Lactobacillus* concentrations and lactate production in the vaginal tract; in the tumor tissue compartment proper, Gram-negative bacterial colonization, inflammation, and lack of bacterial diversity are observed [reviewed in (215)]. Microbial diversity and species richness are reduced in the ovarian microbiota of ovarian cancer tissue compared to distal Fallopian tube samples from healthy patients (218). Specifically, the ratio of *Proteobacteria* to *Firmicutes* is increased in ovarian cancer tissues; significantly elevated *Acinetobacter* and depleted *Lactococcus* are also characteristic of ovarian cancer (218). Banerjee et al. identified an oncobiotic ovarian tumor signature with the predominant phyla being *Proteobacteria* and *Firmicutes* (219). Taken together, these primary studies suggest that FRT microbiome profiling may prove useful as a biomarker of ovarian cancer (163). As for non-malignant disease of the ovaries, PCOS patients have reduced *Lactobacillus* concentrations and increased abundance of non-*Lactobacillus* taxa in the cervical and vaginal environments compared to healthy controls. Furthermore, pathways for antigen processing and presentation, in addition to antibiotic biosynthesis, are overactive in PCOS patients. These findings suggest that PCOS patients have polymicrobial microbiota and inflammation of the lower genital tract (220), and that bacterial perturbances may be involved in ovarian cancer and PCOS.

Although still a relatively new area of research, an increasing body of literature now reports alterations in the FRT microbiotas associated with a range of gynecological conditions including STIs, infertility, endometriosis, and gynecologic cancer. Future studies should aim to characterize the precise mechanisms governing these associations and consider how the information gleaned might be used for diagnostic and therapeutic purposes.

## Diagnostics and therapeutics

The FRT microbiota may be a promising diagnostic and therapeutic target for different gynecological conditions and malignancies (221). The homeostatic interplay between the FRT microbiotas and host immune system may help prevent the development of dysbiosis-associated infections. The changes in the immune and metabolic signaling that take place during dysbiosis could affect the pathophysiology of cancer, such as epithelial barrier breach, angiogenesis, changes in cellular proliferation and apoptosis, and genome instability, ultimately leading to gynecological cancer. For example, there is an association between the vaginal microbiota and vaginal intraepithelial neoplasia (VAIN), more specifically the change in the composition of the vaginal microbiota with an increased abundance of *Atopobium, Gardnerella, Enterococcus, Clostridium and Allobaculum* and a higher viral load of HPV-16, 52, and 58 contribute to the progression of VAIN and growth of vaginal cancer (222). Studies have found specific microbiota characteristics and signatures that are not only potential diagnostic markers for gynecological cancers, but also therapeutic targets. These microbiota characteristics are different in the healthy compared to the cancerous state (163). For example, *Atopobium* and *Porphyromonas* found in the endometrium are associated with endometrial cancer *via* pro-inflammatory cytokine and ROS production, leading to inflammation and increased cell permeability (5, 65).

### Diagnostics

An interesting clinical application for the resident microbiotas in the FRT is its diagnostic potential. The human microbiome could be the second human genome; a study in China used the Vaginal Microecology Evaluation System (VMES) as a tool for analyzing the vaginal microbiome (223). VMES is mostly made of functional and morphological microecological indicators. Functional indicators show the microbial functional status and the activity of the several enzymes, such as β-glucuronidase and acetylglucosaminidase (224). VMES helps evaluate the vaginal ecosystem which helps clinicians diagnose and improve treatment regimens for vaginal infectious diseases.

Additionally, it has been proposed that the FRT microbiotas can be used to diagnose gynecological conditions, such as endometriosis. Perrotta et al. collected vaginal and rectal samples from 35 women with and without endometriosis at two different periods of the menstrual cycle (225). Gut and vaginal microbiotas from patients with different revised American Society for Reproductive Medicine (rASRM) endometriosis stages were analyzed and rASRM stage I–II patients could be differentiated from stages III-IV using the relative abundance of an *Anaerococcus* operational taxonomic unit (225). Another study investigated the effect of endometriosis on uterine and cervical bacterial communities (196). Uterine washes and urogenital swabs were collected from women undergoing surgery for benign uterine/ovarian conditions or for pelvic pain and suspected endometriosis. The bacterial community composition significantly differed between the cervical and uterine samples, and stage III endometriosis samples had a significantly altered cervical bacterial community (196). These findings could serve as a foundation for investigating the role of FRT microbiotas in pathogenesis and diagnosis of endometriosis and other gynecological conditions. However, future research should focus on achieving external validation by replicating these results with larger sample sizes.

The bacterial “signatures” present and/or the immune response to bacterial dysbiosis in the FRT are not only intriguing from an empirical perspective but may also have clinical implications. Although our understanding of the FRT microbiotas, particularly those other than the vaginal microbiota, is still evolving, it is important to think about the potential utility of reported and replicable differences in terms of diagnostics and therapeutics. For example, the immune signatures characterized by Campisciano et al. and Fichorova et al. could prove useful in BV diagnosis, to predict recurrence and assess recovery after treating a FRT infection (156, 162). In another study, Masson et al. developed and validated a biomarker panel able to identify women with asymptomatic vaginal dysbiosis and STIs that cause vaginal discharge (e.g., *C. trachomatis, N. gonorrhea*, etc.) (176). An analysis of cervicovaginal lavage samples from over 200 HIV-uninfected women identified IL-1β and IFN-γ induced protein (IP)-10 as biomarkers of genital inflammation (226). A validation study by the same authors demonstrated that IL-1α, IL-1β, IP-10 could accurately diagnose BV or intermediate microbiota status with 77% sensitivity and 71% specificity, deemed more accurate than detection *via* clinical symptoms alone. The accuracy of this panel was further increased when combined with vaginal pH measurement. This test could be implemented to refer women for further testing to elucidate the cause of inflammation and to initiate appropriate treatment (176). Furthermore, characterization of the vaginal microbiota will pave the way for therapies including probiotics, prebiotics, antibiotics, and hormonal therapies that can shape the FRT microbiota composition to restore eubiosis (17). For example, Wang et al. identified 34 bacterial genera that can predict POF and propose the use of probiotics to re-establish vaginal eubiosis in POF, with the goal of improving IVF success rates among this population (187).

### Probiotics

In addition to the diagnostic potential of the FRT microbiotas, their therapeutic manipulation could offer additional methods to treat and/or manage gynecological conditions. Probiotics are defined as live microorganisms that confer a health benefit if taken adequately (227). Probiotics are commonly used for prevention or treatment of vaginal disorders. Probiotics mostly include *Lactobacillus* species and their role in vaginal health has been studied extensively. Studies have shown that specific probiotic strains increase lactobacilli counts in healthy women and women with Candida vulvovaginitis (vaginal yeast infection) and/or BV and assists vaginal microbiota in recovering from antibiotics/antifungal treatments (228, 229). Antimicrobial treatment of urogenital infections is not always effective and associated with high recurrence rates (230, 231). Infection recurrence could result from a failure of antimicrobials to eliminate pathogens due to biofilm resistance or because commensal bacteria are depleted by the antimicrobial agents (232, 233). Therefore, probiotics may be useful in replenishing commensal bacteria and decreasing the recurrence rate of infections. It was reported that daily *L. acidophilus* treatment was as effective as antimicrobial treatment (trimethoprim/sulfamethoxazole) in reducing UTIs (234). Another study showed that weekly application of *L. rhamnosus* GR-1 and *L. fermentum* B-54 reduced the recurrence of UTIs from 6 to 1.6 per year, further supporting the potential of probiotics in prevention and treatment of urogenital infections (235). Moreover, when the effect of a probiotic vs. pasteurized yogurt on BV episodes was investigated, a greater reduction in BV episodes (60%) was reported in patients consuming probiotic compared to pasteurized yogurt (25%) (236). However, discrepancies exist between different studies due to suboptimal designs, considerable biases, and small samples size that lack adequate statistical power to detect between-group differences (237). Probiotics can have beneficial effects through several mechanisms that include production of hydrogen peroxide [as previously discussed (29)] and lactic acid that lower the vaginal pH; producing antimicrobial compounds and stimulating immune response to help maintain eubiosis in the vaginal tract; and competing for nutrients and restricting pathogen growth by adhering to vaginal epithelial cells (12, 238–241). Moreover, lactic acid also exerts anti-inflammatory effects in cervicovaginal epithelial cells by producing the anti-inflammatory cytokine IL-1RA which inhibits the production of inflammatory mediators IL-6, TNF-α, RANTES, IL-8, and MIP3α from epithelial cell lines and prevents IL-6 and IL-8 production by seminal plasma (242).

Moreover, *Lactobacillus* or *Bifidobacterium* dominant uterine microbiota seems to be associated with better IVF outcomes compared to other microbial profiles (63, 186). This could be due to bacterial modification of inflammation and host immune cell subsets that might be needed for embryo implantation (243). Thus, animal models have been inoculated with various bacterial strains in the uterus and the vaginal tract to examine their effects on fertility. As beneficial reproductive effects of lactobacilli are more commonly reported than *Bifidobacterium*, most studies focus on strains of lactobacilli. The intrauterine administration of *L. buchneri* was associated with improved reproductive performance in cows and reduced inflammatory cytokines (244), while vaginal inoculation with *L. plantarum* restored fertility in mice that had previously been inoculated with a sperm agglutinating strain of *E. coli* (245, 246). Additionally, vaginal treatment with *L. plantarum* was able to negate infertility caused by the intravaginal administration of LPS, indicating that probiotics can ameliorate inflammation-associated infertility, at least in a mouse model (247). Importantly, no toxic effects were reported for the oral administration of *L. helveticus* at doses up to 2000mg/kg with respect to fertility, embryonic, or fetal development in rats (248). With respect to *Bifidobacterium*, oral administration of *B. longum* in mice improved experimentally induced BV by reducing inflammatory factors in the vagina, uterus, and gastrointestinal tract (249). Taken together, these experimental studies suggest that *Lactobacillus* and *Bifidobacterium*-containing probiotics may have the potential to improve fertility by modifying the uterine environment. There are currently three reports of probiotic use during human IVF cycles in the scientific literature. In the first study, the intravaginal administration of probiotics (human strains of *L. acidophilus, B. bifidum* and *B. longum*) did not improve pregnancy rate in women undergoing IVF (250), however a commentary on the topic of probiotics and IVF suggested that the particular probiotic employed in this study may not have been able to produce the desired effect (251). Two subsequent studies, however, were able to demonstrate a positive effect of probiotics on pregnancy outcomes during IVF. Irollo et al. found that 2 months of oral prebiotic-probiotic treatment (type of probiotic and regimen not mentioned in manuscript) prior to frozen embryo transfer (FET) significantly increased the ongoing pregnancy rate, and live birth rate in women with intestinal dysbiosis as compared to women not receiving the treatment (252). Kyono et al. describe a case series of 9 women with non-*Lactobacillus* dominant endometrial microbiota who were treated prophylactically with oral antibiotics in combination with vaginal pre/probiotics (186). The uterine microbiota of all 9 women became *Lactobacillus* dominant following treatment, and 5 of them became pregnant following FET (186). Taken together, early studies suggest a potential therapeutic role for probiotics in FRT health and fertility.

### Vaginal microbiome transplant (VMT)

Other than probiotics, another potential avenue to modify the vaginal microbiota is *via* VMT. Interest in using transplanted human material, more specifically vaginal microbiome for microbial infection and dysbiosis, started after the success of fecal microbiota transplantation for treating recurrent *Clostridium difficile* infection (253). Additionally, vaginal microbiome components have been found to transfer between women who have sex with women based on a study that showed 23 out of the 31 monogamous female couples had identical rep-PCR fingerprints of *Lactobacillus* spp. (254). VMT from a donor with an “optimal” vaginal microbiota has been investigated as a potential therapy for women with BV or other vaginal disorders. Lev-Sagie et al. showed that VMT from healthy donors attenuated BV in four out of five participants with intractable, symptomatic, and recurrent BV (255). These women showed improvement in symptoms, reconstitution of a *Lactobacillus*-dominated VMB, and appearance of vaginal fluid on a microscopic level. This lasted 5–21 months where the four patients did not need repeated VMT or additional treatments (255). The fifth subject also showed partial improvement of symptoms and expansion of *Lactobacillus* species (255). There are some risks associated with VMT, such as transfer of antimicrobial-resistant microorganisms and pathogens from donor to recipient, unintentional sperm transfer which could result in unintended pregnancy, transfer of other phenotypes and infectious diseases that may remain undetected using current screening methods, and immunologic rejection (256, 257). In March 2020, a Safety Alert regarding the potential for transmission of SARS-CoV2 *via* fecal matter in Fecal Microbiota Transplantations was issued by the Food and Drug Administration which may suggest a similar transmission potential in VMTs (258). Therefore, having strict inclusion and exclusion criteria and extensive testing of the donor samples is necessary to minimize risks associated with VMT (259). Currently there are two ongoing clinical trials investigating the use of VMTs, one in Massachusetts General Hospital and another at Johns Hopkins (260, 261). However, to the best of our knowledge the only published research on VMTs is the Lev-Sagie et al. study (255). Therefore, future research with bigger sample size is required to further investigate the safety, effectiveness, and durability of VMTs.

### Antibiotics

Antibiotics are the first line therapy for BV (262). These antibiotics include metronidazole, clindamycin, and tinidazole that could be administered orally and/or topically. Metronidazole is a nitroimidazole antimicrobial agent which is used for managing protozoal infections such as anaerobic infections and trichomoniasis and could be administered orally or vaginally (263). Studies have shown that the cure rates of women taking metronidazole for BV were between 58–100%, which was higher than women taking placebo (5–29%) (264, 265). Clindamycin is another antimicrobial agent used to treat BV. This antibiotic is a subclass of the larger family of macrolide antibiotics and could be used orally and vaginally. A meta-analysis determined that clindamycin had a lower treatment failure compared to the placebo (265). Tinidazole is a nitroimidazole antibiotic and was first reported to be used for BV treatment in Europe, Asia, and Latin America (266). The effectiveness of tinidazole was assessed in a randomized control trial and a higher cure rate (dose-dependent) in the tinidazole group compared to the placebo was reported (267).

Although antibiotics are effective against BV, they are associated with a high recurrence rate (50%−67%) and many patients relapse after the treatment (86, 268, 269). This high recurrence rate could be due to reinfection from sexual partners, or it could be because antibiotics are not able to eliminate the biofilm-associated bacteria of BV in the vagina (270–275). For example, it was shown that after treatment with metronidazole the BV-associated bacteria were largely depleted or fully depleted in 58% of patients, possibly because the BV-associated bacteria were sheltered by biofilms (273). Another study showed that *G. vaginalis* biofilms persisted after the oral administration of metronidazole (271). Antibiotics may not always be optimal in treating BV due to resistance from bacteria and fungi which could be a naturally acquired resistance or due to biofilm formation (270, 276–278). Antibiotics could also lead to BV by interrupting endogenous lactobacilli (274, 279). Another pitfall of antibiotics is that they are not bacterial species-specific and could deplete the commensal bacteria, and result in a decrease in the “healthy” bacteria in the vaginal tract (280).

Interestingly, antibiotics have also been reported to have potential therapeutic benefit in animal models for other gynecologic conditions like endometriosis. Mice with surgically induced endometriosis were treated with broad-spectrum antibiotics or metronidazole; endometriotic lesions were significantly smaller after 21 days with fewer proliferating cells in mice treated with antibiotics compared to vehicle (193). Moreover, mice treated with antibiotics showed a significant reduction in inflammatory responses which was measured by the concentration of IL-1β, IL-6, TNF-α, and TGF-β1 in peritoneal fluid and the macrophage marker Iba1 in lesions (193). These findings suggested the microbiotas associated with endometriosis promoted its progression, but further research is needed to evaluate whether these findings can be translated to humans and if new microbiota-based therapies could be developed to manage gynecological conditions or serve as an adjuvant to current therapies.

## Conclusion

The FRT, similar to other mucosal sites, has a site-specific microbiota that forms a mutualistic relationship with human host and plays a critical role in health and maintaining homeostasis. The vaginal microbiota is well-characterized, and it was previously accepted that distal sites of the FRT were sterile. In fact, the endometrial microbiota has been characterized in several studies and shown to vary in composition in women prone to adverse reproductive outcomes or benign/malignant diseases of the endometrium (4, 6, 188, 203). Recent studies have shown specific patterns of microbiota in the upper reproductive tract (uterus, Fallopian tubes, ovaries, and placenta) and have further contested the sterile assumption (7, 23, 67, 73, 74, 79, 83). However, the upper FRT microbiotas are understudied due to complex and invasive sample collection; thus, results of these early studies require confirmation.

**Figure 3:**
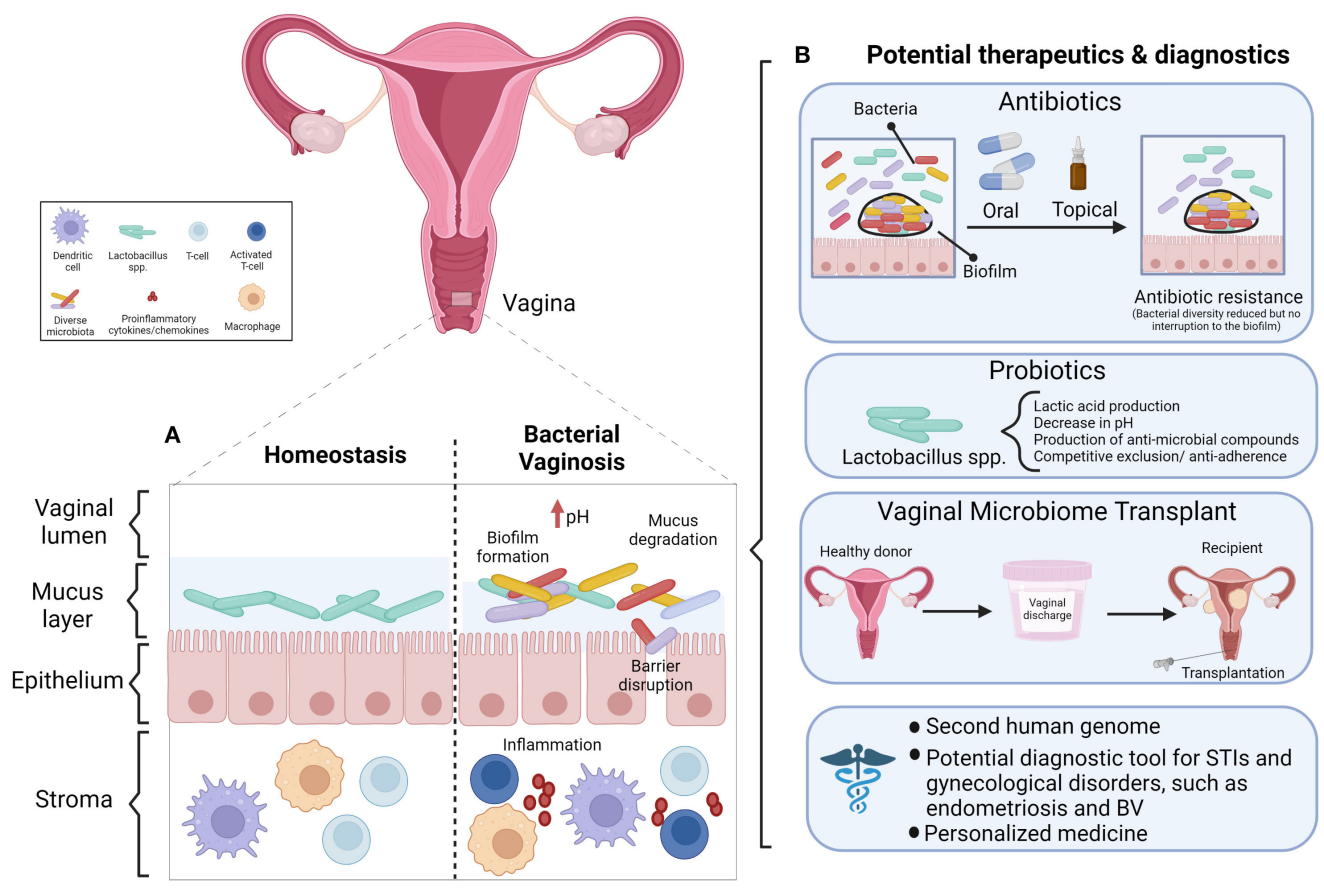
Summary of the female reproductive tract microbiota, inflammation, and therapeutics and diagnostics. **(A)** An illustration depicting a summary of the vaginal microbiota in homeostasis vs. a dysbiotic state (as in Bacterial vaginosis). In homeostasis, the native bacteria are tolerated by the host immune system, while in a dysbiotic state the bacteria and/or metabolites induce an immune response and inflammation in the host. Barrier disruption can occur as a result. **(B)** An overview of how the female reproductive microbiotas might be harnessed as potential therapeutics and diagnostics for gynecological conditions involving inflammation and/or dysbiosis in the female reproductive tract. BV, Bacterial vaginosis; STI, sexually transmitted infections. Created with BioRender.com.

The interaction between the FRT microbiotas and the immune system is complex and necessary for maintaining homeostasis and preventing infection and dysbiosis (Figure 3A). However, to the best of our knowledge there are currently no studies on the mechanism through which the commensal bacteria and immune system interact in FRT. Based on the commensal bacteria-host interactions in other bodily systems, such as the gastrointestinal tract (99–101), it is likely that under normal circumstances, such as healthy diet and lifestyle, eubiosis is maintained through regulatory T cells. However, under certain conditions such as stress and infection this balance can be disturbed, resulting in inflammation and dysbiosis. The literature discussed herein demonstrates that the FRT microbiotas likely play a role in gynecologic conditions, however whether microbial dysbiosis leads to or is a result of gynecological disease has yet to be elucidated. Whether malignant or non-malignant, a consistent pattern emerges from the literature: *Lactobacillus* depletion and increased abundance of “harmful” bacterial species appear to be associated with disease of the FRT. There is a well-established link between dysbiosis and infertility or adverse pregnancy outcome. Furthermore, patients with gynecologic conditions including endometriosis and PCOS often experience infertility. Both conditions are associated with increased presence of pathogenic microbial species throughout the FRT (6, 194, 220). An interesting avenue for future research would be to investigate differences in FRT composition among women with gynecologic disease, in association with prospective infertility and reproductive outcomes in the same population. The FRT microbiotas may be promising for diagnostic and therapeutic purposes (Figure 3B). As discussed herein, the FRT microbiotas can be modified using probiotics, VMTs, and antibiotics. Some studies showed that endometriosis stages could be differentiated using FRT bacteria (225). Moreover, probiotics can re-establish vaginal eubiosis in POF (187) and treating mice with surgically induced endometriosis with antibiotics reduces lesion size (193). Therefore, microbiome-targeted interventions could serve as preventive tools for gynecological conditions in the future. Additional studies should investigate the FRT microbiotas to gain mechanistic insights for personalized medicine and diagnostics.

## Author contributions

MG and EA-D wrote the manuscript and developed visualizations. JW supervised the project. All authors outlined the content of the review, reviewed, edited, and approved the final draft.

## Conflict of interest

Authors JW is Co-founder and Chief Scientific Officer and EA-D is a Product Manager at AIMA Laboratories Inc., a company with products designed to address non-malignant gynecological issues in Women's Health. The remaining author declares that the research was conducted in the absence of any commercial or financial relationships that could be construed as a potential conflict of interest.

## Publisher's note

All claims expressed in this article are solely those of the authors and do not necessarily represent those of their affiliated organizations, or those of the publisher, the editors and the reviewers. Any product that may be evaluated in this article, or claim that may be made by its manufacturer, is not guaranteed or endorsed by the publisher.
